# Cariprazine in Pediatric Patients with Autism Spectrum Disorder: Results of a Pharmacokinetic, Safety and Tolerability Study

**DOI:** 10.1089/cap.2022.0097

**Published:** 2023-08-16

**Authors:** Paul P. Yeung, Kimball A. Johnson, Robert Riesenberg, Amelia Orejudos, Todd Riccobene, Hari V. Kalluri, Paul R. Malik, Shane Varughese, Robert L. Findling

**Affiliations:** ^1^ Clinical Development Neuroscience, AbbVie Inc., Madison, New Jersey, USA.; ^2^ iResearch, Decatur, Georgia, USA.; ^3^ Atlanta Center for Medical Research, Atlanta, Georgia, USA.; ^4^ Department of Psychiatry, Virginia Commonwealth University, Richmond, Virginia, USA.

**Keywords:** cariprazine, pediatric, pharmacokinetic, atypical antipsychotic, autism spectrum disorder

## Abstract

**Objective:**

Cariprazine is a dopamine D_3_-preferring D_3_/D_2_ and serotonin 5-HT_1A_ receptor partial agonist approved to treat adults with schizophrenia and manic/mixed or depressive episodes associated with bipolar I disorder. This study, which is the first to evaluate cariprazine in pediatric patients with autism spectrum disorder (ASD) (including children 5–9 years of age) using an oral solution formulation, evaluated the safety, tolerability, pharmacokinetics (PK), and exploratory efficacy of cariprazine and its two major active metabolites, desmethyl cariprazine (DCAR) and didesmethyl cariprazine (DDCAR).

**Methods:**

This clinical pharmacology, open-label, multiple-dose study enrolled 25 pediatric patients from 5 to 17 years of age, who met the Diagnostic and Statistical Manual of Mental Disorders, Fifth Edition criteria for ASD. All patients began treatment with cariprazine 0.5 mg once daily (QD) and underwent a titration over 7 days to maintenance doses of 1.5 or 3 mg QD for patients 13–17 years of age at Screening, 0.75 or 1.5 mg QD for patients 10–12 years of age at Screening, and 0.5 or 1.5 mg QD for patients 5–9 years of age at Screening. After 6 weeks total of dosing, there was a 6-week follow-up period. Study assessments included adverse events (AEs), safety parameters, noncompartmental PK parameters, and exploratory efficacy assessments, including the Aberrant Behavior Checklist-Irritability Subscale (ABC-I), Clinical Global Impressions (CGI-S), Caregiver Global Impressions (CgGI-S), Children's Yale-Brown Obsessive Compulsiveness Scale Modified for ASD (CYBOCS-ASD), Social Responsiveness Scale (SRS), and Vineland Adaptive Behavior Scale (VABS-III).

**Results:**

All AEs were mild or moderate in severity. Most frequent treatment-emergent adverse events (TEAEs) were increased weight, increased alanine aminotransferase, increased appetite, dizziness, agitation, and nasal congestion. Increases in weight were not considered clinically meaningful. Two subjects reported extrapyramidal symptom-related TEAEs that resolved without leading to discontinuation. Dose-normalized exposures of all analytes were modestly higher in pediatric patients from 5 to 9 years of age when compared to older patients. Consistent with previous studies, at steady state, the rank of exposure in plasma was DDCAR > cariprazine > DCAR. There was numerical improvement on all exploratory endpoints (ABC-I, CGI-S, CgGI-S, CYBOCS-ASD, SRS, and VABS-III).

**Conclusions:**

PK of cariprazine and its metabolites were characterized in pediatric patients with ASD at doses up to 3 mg QD (13–17 years) and 1.5 mg QD (5–12 years). Caripazine treatment was generally well tolerated and results from this study will inform the selection of appropriate pediatric doses for subsequent studies.

## Introduction

Cariprazine is an orally active and potent dopamine D_2_ and D_3_ receptor partial agonist with preferential binding to D_3_ receptors and partial agonist activity at serotonin 5-HT_1A_ receptors. In randomized, double-blind, placebo-controlled trials, cariprazine demonstrated efficacy versus placebo in adults with schizophrenia (Durgam et al. 2014, 2015b; Kane et al. 2015) and in adults with manic/mixed episodes (Calabrese et al. 2015; Durgam et al. 2015a; Sachs et al. 2015) or depressive episodes (Durgam et al. 2016; Earley et al. 2019, 2020) associated with bipolar I disorder. To date, cariprazine has been approved for the treatment of schizophrenia in adults, as well as for manic, mixed, and depressive episodes associated with bipolar 1 disorder in adults, in the United States, the European Union, and other countries at doses of 1.5–6 mg/day.

In adults, cariprazine displays dose-proportional pharmacokinetics (PK) and follows a triphasic elimination curve (Periclou et al. 2021). Following oral administration, cariprazine is efficiently absorbed, but achieves incomplete bioavailability due to first-pass metabolism. Cariprazine is mainly metabolized by CYP3A4 and, to a lesser extent, by CYP2D6 to two active metabolites, desmethyl cariprazine (DCAR) and didesmethyl cariprazine (DDCAR), which possess similar pharmacological activity to the parent drug. At steady state, the rank of exposure in plasma is DDCAR > Cariprazine > DCAR. The three active moieties distribute into tissues, including the brain, owing to their lipophilicity. In adults, the terminal elimination half-lives of cariprazine, DCAR, and DDCAR are 3–4 days, 1–2 days, and 1–3 weeks, respectively. Less than 5% of cariprazine is excreted unchanged in urine or bile.

The pediatric development program for cariprazine is ongoing, and requires information on the PK, safety, and tolerability of cariprazine in pediatric patients to inform dose selection. Previous studies were conducted in pediatric patients from 10 to 17 years with bipolar I disorder and from 13 to 17 years with schizophrenia to assess PK, safety, and tolerability following administration of cariprazine capsules (Riccobene et al. 2022). Dosing was initiated at 0.5 mg or 1.5 mg/day, followed by titration every 2–3 days up to as high as 4.5 mg/day. In these patient populations, cariprazine displayed a similar PK profile to that observed in adults and was generally safe and well tolerated. Most frequent treatment-related treatment-emergent adverse events (TEAEs) included sedation, Parkinsonism, tremor, dystonia, and blurred vision.

Autism spectrum disorder (ASD) is a prevalent neurodevelopmental condition with symptoms in early childhood, although the disorder may first be diagnosed later in life. There is currently no medication specifically approved in the United States for the treatment of the core features of ASD (social withdrawal, stereotypical behavior, and others). Atypical antipsychotics are shown to be safe and efficacious in the treatment of irritability associated with ASD. Based on the known pharmacological profile of cariprazine and its similarity to antipsychotics, cariprazine could be effective in treating ASD-associated irritability and/or core symptoms.

The exact pathogenesis of ASD has not yet been discovered, but there is some evidence to indicate that the central dopaminergic system is implicated in ASD. Variants of the D_1_, D_2_, D_3_, and D_4_ dopamine receptor genes have been associated with ASD (Hettinger et al. 2012; Nguyen et al. 2014). The dopamine D_3_ receptor gene is highly expressed in the basal ganglia and D_3_ receptor stimulation plays a role in this cortical dopaminergic hypofunctionality; hence, it may be the neurochemical basis impacting social behaviors and cognitive functions. It is of particular interest that a dopamine D_3_ receptor single nucleotide polymorphism (rs167771) was associated with ASD in multiple independent populations and positively correlated with stereotyped behavior and larger striatal volume (de Krom et al. 2009; Staal 2015; Staal et al. 2012).

A novel oral solution formulation has been developed to support the investigation of cariprazine in pediatric patients with ASD down to 5 years of age, who may have difficulty swallowing capsules. As an important step in the pediatric development program, this study represents the first investigation of the PK, safety, and tolerability following administration of cariprazine as an oral solution formulation in pediatric patients with ASD down to 5 years of age. In addition, the efficacy of cariprazine for the treatment of irritability associated with ASD and/or the core symptoms of ASD was examined as an exploratory outcome using a battery of clinical rating scales.

## Materials and Methods

### Study participants

This study enrolled 25 male and female pediatric patients from 5 to 17 years of age with ASD, according to criteria in the Diagnostic and Statistical Manual of Mental Disorders, Fifth Edition (APA 2013). At the first visit, the study was discussed with each participant and with the participant's parent or legally authorized representative, and written informed consent was obtained. Participants with other psychiatric or mental disorders, ophthalmological abnormalities, or clinical neutropenia (absolute neutrophil count [ANC] ≤1 × 10^9^ cells/L), or who displayed suicidal or homicidal attempt or intent in the 6 months before screening were excluded. Participants who previously received cariprazine or those who needed concomitant treatment with moderate or strong CYP3A4 inhibitors or CY3A4 inducers were also excluded. Full inclusion and exclusion criteria are provided in Tables 1 and 2, respectively.

**Table 1.** Inclusion Criteria—Participants Are Eligible to Be Included in the Study Only If All the Following Criteria Apply

1.	Age
1.01	Participant must be 5–17 years of age, inclusive, at the time of signing the informed consent and assent (assent will be obtained if age appropriate according to the IRB)
2.	Type of participant and characteristics
2.01	Participants must meet the DSM-5 criteria for ASD diagnosis
2.02	Participants must have normal physical examination findings and clinical laboratory test results for their age group or abnormal results judged not clinically significant by the investigator
2.03	Negative serum hCG pregnancy test at screening (all female participants who have reached menarche)
3.	Weight and BMI
3.01	BMI greater than the 5th percentile for age and gender based on CDC growth charts
4.	Sex
4.01	Male or female
5.	Contraceptives
5.01	Participant (if reached his spermarche or her menarche) must agree to sexual abstinence or to use an approved birth control method for the full duration of participation in the study. The investigator and each participant will determine the appropriate method of contraception for the participant during their participation in the study
6.	Informed consent
6.01	Participant's parent(s)/legally authorized representative must be capable of giving signed informed consent, which includes compliance with the requirements and restrictions listed in the ICF and in this protocol, as explained by the investigator. Written informed consent from the participant's parent(s)/legally authorized representative must be obtained before any study-related procedure
6.02	Assent (if age appropriate according to the IRB) must be obtained for all participants participating in the study
7.	Other
7.01	As assessed by the investigator, participant must be able and willing to follow study instructions and likely to complete all required study visits
7.02	Participant must have parent(s) or legally acceptable representative who is willing and able to be responsible for safety monitoring of the participant, provide information about the participant's condition, oversee administration of study intervention, and accompany the participant to all study visits. The caregiver can be the participant's parent(s)/legally authorized representative. Written consent from the caregiver must be obtained

ASD, autism spectrum disorder; BMI, body mass index; CDC, Center for Disease Control and Prevention; DSM-5, Diagnostic and Statistical Manual of Mental Disorders, Fifth Edition; hCG, human chorionic gonadotrophin; ICF, informed consent form; IRB, institutional review board.

**Table 2.** Exclusion Criteria—Participants Are Excluded from the Study If Any of the Following Criteria Apply

1.	Medical conditions
1.01	Current diagnosis of bipolar disorder, schizophrenia, schizoaffective disorder, schizophreniform disorder, brief psychotic disorder, or psychotic disorder due to another medical condition
1.02	Diagnosis of intellectual disability (IQ <70)
1.03	Participant has a history of meeting DSM-5 diagnosis for any substance-related disorder (except caffeine and tobaccorelated) within the 3 months before the Screening Visit
1.04	Participant with an acute or unstable medical condition, including (but not limited to) inadequately controlled diabetes, hepatic insufficiency (specifically any degree of jaundice), uncorrected hyperthyroidism or hypothyroidism, acute systemic infection, renal, gastrointestinal, respiratory, or cardiovascular disease
1.05	Any clinical condition or previous surgery that might affect the absorption, distribution, biotransformation, or excretion of cariprazine
1.06	History of seizures, with the exception of febrile seizures
1.07	History of tumor of the central nervous system
2.	Prior/concomitant therapy
2.01	Previously taken cariprazine or previously participated in an investigational study of cariprazine
2.02	Participant requires concomitant treatment with moderate or strong CYP3A4 inhibitors or CYP3A4 inducers
2.03	Participant requires concomitant treatment with any prohibited medication, with the exception of permitted interventions
2.04	Use of an antipsychotic depot within 2 cycles of their respective dosing interval before the Screening Visit
2.05	Participant is unwilling to discontinue or, in the opinion of the investigator, unable to safely taper off any protocol-specified prohibited treatment before study day 1 without significant destabilization or increased suicidality
3.	Prior/concurrent clinical study experience
3.01	Participant is currently enrolled in an investigational drug or device study or participation in such a study within 3 months of study day 1
3.02	Participation in a blood or plasma donation program within 60 or 30 days, respectively, before study day 1
4.	Diagnostic assessments
4.01	Out of range values for sitting systolic or diastolic BP are sitting systolic BP >125 mm Hg or <85 mm Hg or sitting diastolic BP >80 mm Hg or <55 mm Hg at the Screening Visit
4.02	Out of range values for sitting PR/HR are <60 and >20 bpm for all participants during the vital sign assessment at the Screening Visit
4.03	Abnormal ECG results thought to be potentially clinically significant according to the investigator or designee, or QT prolongation (QTcB ≥460 msec) at the Screening Visit
4.04	Positive UDS for substances of abuse at the Screening Visit or on study day −1. Exception: participants with a positive UDS at screening for opiates, cannabinoids, amphetamines, or benzodiazepines may be allowed in the study, provided all 3 of the following apply: a. The drug was used for a legitimate medical purpose with the exception of poppy seed consumption; b. The drug can be discontinued before further participation in the study (except for benzodiazepines, which may be continued if the participant has been taking a stable dose [ie, lorazepam up to 2 mg/day or its benzodiazepine equivalent] for at least 1 month prior the Screening Visit or if used as rescue during washout); and c. A repeat UDS is negative for these substances before study day 1, except benzodiazepine use as described above
4.05	Participants who meet any of the following ophthalmological criteria: a. A finding of cataracts (lens opacifications) at the screening ocular examination. b. Any clinically significant ocular trauma or complications of ocular trauma, or history of retinal detachment, intraocular surgery, or laser treatment. c. History or current findings of ocular disease (open- or narrow-angle glaucoma, retinopathies, or corneal diseases). d. History of amiodarone or systemic corticosteroid use for ≥3 consecutive months in the past year. e. Intraocular pressure of >21 mm Hg in either eye
4.06	Absolute neutrophil count ≤1.0* ×* 10^9^/L at the Screening Visit
4.07	Screening liver enzyme test (AST and/or ALT) results >2* ×* ULN or total bilirubin >1* ×* ULN
4.08	Positive test results for HIV types 1 and 2 antibody, hepatitis B surface antigen, or anti-hepatitis C virus antibody at the Screening Visit
5.	Other exclusions
5.01	For female participants, positive serum pregnancy test at the Screening Visit, positive serum or urine pregnancy test on study day −1, or nursing, or planning a pregnancy at any time during participation in the study
5.02	Known allergy or sensitivity to the study intervention or its components
5.03	History of serious homicidal risk or behavior that resulted in hospitalization or adjudication (legal sentencing) within 6 months of the Screening Visit
5.04	Any current suicidal ideation or a history of active suicidal ideation within the past 6 months or suicide attempts within the past year
5.05	The participant has a condition or is in a situation which, in the investigator's opinion, may put the participant at significant risk, may confound the study results, or may interfere significantly with the participant's participation in the study
5.06	Consumption of grapefruit, grapefruit juice, vegetables from the mustard green family (e.g., kale, broccoli, watercress, collard greens, kohlrabi, brussels sprouts, mustard), or charbroiled meats within 72 hours before administration of study intervention
5.07	Consumption of caffeine- or xanthine-containing products (including, but not limited to coffee, tea, caffeinated soft drinks, energy/sports drinks, and chocolate), exceeding 200 mg of caffeine or 4 oz. of chocolate per day within 48 hours of study intervention administration
5.08	Consumption of alcohol within 72 hours before administration of study intervention
5.09	Directly or indirectly involved in the conduct and administration of this study as an investigator, subinvestigator, study coordinator, or other study staff member; or employee of the sponsor or a first-degree family member, significant other, or relative residing with one of the above persons involved directly or indirectly in the study

ALT, alanine aminotransferase; AST, aspartate transaminase; BP, blood pressure; DSM-5, Diagnostic and Statistical Manual of Mental Disorders, Fifth Edition; ECG, electrocardiogram; HR, heart rate; IQ, intelligence quotient; PR, pulse rate; QTcB, QTC corrected by Bazett's; UDS, urine drug screen; ULN, upper limit of normal.

### Study design

This clinical pharmacology, open-label, multiple-dose study evaluated the PK, safety, tolerability, and exploratory efficacy of cariprazine in pediatric patients with ASD. The study protocol and all other study-related documents were reviewed and approved by an ethics committee/independent institutional review board (IRB) at each participating site. All subjects provided written, informed consent prior to the start of any screening or study-specific procedures. The study was conducted at three investigative sites in the United States from June 26, 2020, to December 10, 2021 in conformance with the International Council for Harmonisation E6 guideline for Good Clinical Practice and the principles of the Declaration of Helsinki.

Patients were screened for up to 28 days before dosing, and previous antipsychotics or mood stabilizers were discontinued during a washout period of 4–7 days before dosing. Patients received cariprazine during a 42-day treatment period, but did not receive cariprazine and could take prior medication during a 42-day posttreatment PK and safety follow-up period. Participants were required to be inpatient during the initiation of study treatment (days −1 to 7). After day 7, the participant could remain an inpatient or continue treatment as an outpatient, based on clinical judgment of the site investigator. Participants were required to be inpatient from day 41 until collection of the 24-hour blood sample on day 43.

Participants were assigned to 1 of 4 cohorts according to age and maintenance dose of cariprazine. Enrollment of Cohorts 2 and 3 began after a data monitoring committee (DMC) reviewed safety, tolerability, and PK data from Cohort 1. Cohorts 2 and 3 were initiated based on results from Cohort 1 and were enrolled in parallel. Enrollment of Cohort 4 began after the DMC reviewed safety, tolerability, and PK data from Cohorts 1, 2, and 3. All patients began treatment with cariprazine 0.5 mg once daily (QD) in an oral solution formulation and underwent a titration over 7 days to maintenance doses of 1.5 or 3 mg QD for patients 13–17 years of age at screening, 0.75 or 1.5 mg QD for patients 10–12 years of age at screening, and 0.5 or 1.5 mg QD for patients 5–9 years of age at screening (Table 3).

**Table 3.** Summary of Dosing and Up-Titration Schedules for Cohorts 1 to 4 (Pediatric Participants with Autism Spectrum Disorder)

Intervention/postintervention day	10–17 years				5–9 years	
	Cohort 1		Cohort 2		Cohort 3	Cohort 4
	10–12 years, 0.75 mg/day	13–17 years, 1.5 mg/day	10–12 years, 1.5 mg/day	13–17 years, 3.0 mg/day	0.5 mg/day	1.5 mg/day
	*N* = 6		*N* = 6		*N* = 6	*N* = 6
Day 1, mg (mL)	0.5 (1)	0.5 (1)	0.5 (1)	0.5 (1)	0.5 (1)	0.5 (1)
Day 2, mg (mL)	0.5 (1)	0.5 (1)	0.5 (1)	0.5 (1)	0.5 (1)	0.5 (1)
Day 3, mg (mL)	0.5 (1)	0.5 (1)	0.5 (1)	0.5 (1)	0.5 (1)	0.5 (1)
Day 4, mg (mL)	0.75 (1.5)	1.5 (3)	1.5 (3)	1.5 (3)	0.5 (1)	1.5 (3)
Day 5, mg (mL)	0.75 (1.5)	1.5 (3)	1.5 (3)	1.5 (3)	0.5 (1)	1.5 (3)
Day 6, mg (mL)	0.75 (1.5)	1.5 (3)	1.5 (3)	1.5 (3)	0.5 (1)	1.5 (3)
Days 7–42	0.75 (1.5)	1.5 (3)	1.5 (3)	3.0 (6)	0.5 (1)	1.5 (3)
Days 43–84	PK follow-up	PK follow-up	PK follow-up	PK follow-up	PK follow-up	PK follow-up

PK, pharmacokinetics.

### Sample collection

Whole blood samples of ∼2 mL were collected and processed for measurement of plasma concentrations of cariprazine, DCAR, and DDCAR at the following times: day 1 at predose, and at 2, 3, 4, 6, 8, and 24 hours (i.e., predose on day 2) postdose; days 7, 14, 21, 28, and 35 at predose; and starting on day 42 at predose and at 2, 3, 4, 6, 8, 24 (day 43), 48 (day 44), 168 (day 49), 336 (day 56), 672 (day 70), and 1008 (day 84) hours postdose. Plasma concentrations of cariprazine, DCAR, and DDCAR were determined using a validated liquid chromatography method with tandem mass spectrometric detection. The lower limits of quantitation (LLOQ) were 0.02, 0.02, and 0.05 ng/mL for cariprazine, DCAR, and DDCAR, respectively. Concentrations below the LLOQ were reported as zero.

### Assessments

The following PK parameters were calculated for cariprazine, DCAR, and DDCAR using noncompartmental methods in Phoenix WinNonlin v8.3: maximum observed plasma concentration (C_max_), time to C_max_ (T_max_), area-under-the-curve over the dosing interval (AUC_0-τ_), and terminal elimination half-life following the last dose on day 42 (t_1/2_). In addition, steady-state oral clearance (CL_ss_/F) was determined for cariprazine.

The following safety measurements were performed at each visit during the study: clinical assessment of adverse events (AEs)/serious adverse events (SAEs), measurement of vital signs, physical examinations, 12-lead electrocardiograms (ECGs), clinical safety laboratory assessments, slit-lamp ophthalmologic examinations, and Columbia-Suicide Severity Rating Scale (C-SSRS) (Posner et al. 2011) assessments for suicidality.

To assess extrapyramidal symptoms (EPS), the Abnormal Involuntary Movement Scale (AIMS) (Guy 1976), the Barnes Akathisia Rating Scale (BARS) (Barnes 1989), and the Simpson-Angus Scale (SAS) (Hawley et al. 2003) were assessed at weekly intervals until day 56 and then every 2 weeks until day 84.

Exploratory efficacy assessments included the Aberrant Behavior Checklist-Irritability Subscale (ABC-I), Clinical Global Impressions (CGI-S), Caregiver Global Impressions (CgGI-S), Children's Yale-Brown Obsessive Compulsiveness Scale Modified for ASD (CYBOCS-ASD), Social Responsiveness Scale, 2nd Edition (SRS), and Vineland Adaptive Behavior Scale, 3rd Edition (VABS-III).

### Data analysis

PK parameters were calculated by noncompartmental methods using standard equations in Phoenix WinNonlin v8.3. C_max_ and T_max_ were derived from the data. The AUC_0-τ,ss_ was calculated by using the linear-log trapezoidal rule. Estimates of t_1/2_ were calculated based on λ_z_. The λ_z_ was determined by performing a regression analysis on the terminal linear phase of semilogarithmic plots of individual cariprazine, DCAR, and DDCAR concentration-time data using a minimum of three concentration-time points in the elimination phase, excluding C_max_. λ_z_ was valid when the adjusted *R*^2^ was >0.80.

CL_ss_/F was calculated as: $\frac{Dose}{AUC_{0-\tau ,ss}}$

Dose proportionality within age groups was assessed by contrast in dose-normalized exposures (C_max_ and AUC_0-τ,ss_). The influence of pediatric body weight on cariprazine CL/F was determined by estimating an allometric coefficient (θ) with reference to the typical CL/F in adults (21.5 L/h in a 79-kg adult) (Periclou et al. 2021).

$$CL∕F_{child}=21.5L∕h\times {\left(\frac{BW_{child}}{79kg}\right)}^{\theta }$$

Descriptive statistics were presented for safety parameters and exploratory efficacy parameters, summarized by treatment group. Change from baseline in body weight was evaluated using standardized *z*-score methodology.

## Results

### Patient characteristics

A total of 25 pediatric patients with ASD were enrolled in the study and received at least one dose of cariprazine; overall, 24 (96%) of these patients completed the 6-week treatment period, and 19 (76%) completed both the 6-week treatment period and the 6-week follow-up period.

Patient demographics and baseline characteristics were generally similar between age groups and treatment groups (Table 4). Most patients were Black or African American (72%), and there was an approximately even sex distribution between males (52%) and females (48%).

**Table 4.** Demographic Summary

	Median (mean, SD)	Min–Max
	Cohort 1 (Total* N* = 7) (*N* = 3, 10–12 years;* N* = 4, 13–17 years)	
Age (years)	14.0 (14.3, 2.36)	12–17
Weight (kg)	61.1 (63.1, 19.5)	34.4–92.8
BMI kg/m^2^	21.7 (22.3, 5.24)	16.7–30.1
Sex	4 Males (57%), 3 Females (43%)	
Race	2 White (29%), 5 Black or African-American (71%)	

	Cohort 2 (Total* N* = 6) (*N* = 3, 10–12 years;* N* = 3, 13–17 years)	
Age (years)	12.0 (12.5, 2.26)	10–16
Weight (kg)	52.7 (61.6, 25.7)	40.4–112
BMI kg/m^2^	21.3 (23.7, 8.43)	16.8–39.5
Sex	2 Males (33%), 4 Females (67%)	
Race	1 White (17%), 5 Black African-American (83%)	

	Cohort 3 (*N* = 6)	
Age (years)	8.0 (7.80, 1.20)	6–9
Weight (kg)	37.9 (36.4, 8.74)	23.4–45.5
BMI kg/m^2^	20.1 (20.1, 2.88)	16.0–24.6
Sex	3 Males (50%), 3 Females (50%)	
Race	2 White (33%), 4 Black or African-American (67%)	

	Cohort 4 (*N* = 6)	
Age (years)	7.5 (7.30, 1.60)	5–9
Weight (kg)	25.6 (26.0, 4.19)	21.5–32.3
BMI kg/m^2^	17.0 (18.1, 3.98)	14.5–25.1
Sex	4 Males (67%), 2 Females (33%)	
Race	2 White (33%), 4 Black or African-American (67%)	

BMI, body mass index; Max, maximum; Min, minimum; SD, standard deviation.

### Pharmacokinetics

**FIG. 1.**
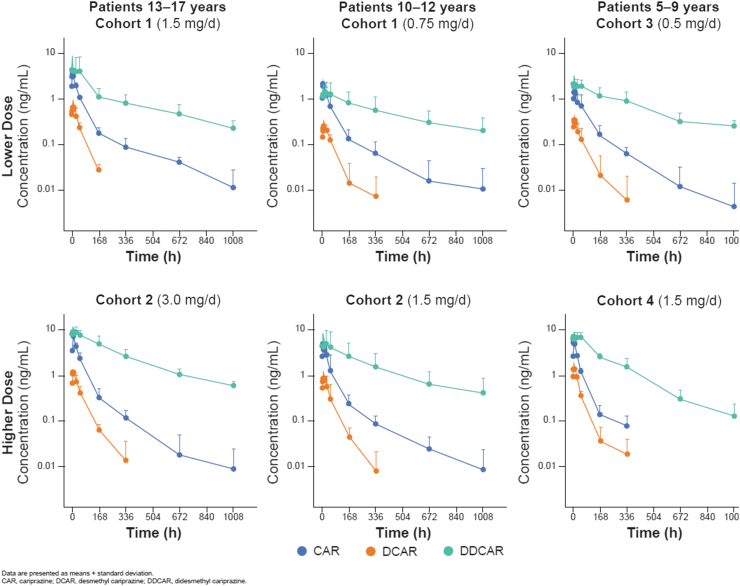
Plasma concentrations of cariprazine and metabolites after dosing on day 42.

First dose and steady-state (day 42) PK of cariprazine, DCAR, and DDCAR were characterized in children 5–17 years of age with ASD at multiple dose levels (Table 5; Fig. 1). Following a single-dose administration of 0.5 mg cariprazine to pediatric patients on day 1, measurements of DCAR plasma concentration were generally low, and DDCAR levels were undetectable; therefore, PK parameters could not be determined on day 1 for these analytes.

**Table 5.** Geometric Mean (Mean, % Coefficient of Variation) Pharmacokinetic Parameters of Cariprazine and Metabolites on Day 42

	Cohort 1		Cohort 2		Cohort 3	Cohort 4
Age (years)	10–12	13–17	10–12	13–17	5–9	5–9
Dose (mg)	0.75	1.5	1.5	3.0	0.5	1.5
*N*	3	3	3	3	6	6
Cariprazine						
C_max,ss_ (ng/mL)	2.13 (2.17, 21.4)	3.66 (3.77, 28.5)	5.04 (5.42, 48.7)	9.42 (9.44, 7.15)	1.74 (1.84, 39.2)	6.40 (6.49, 19.2)
T_max_ (h)^a^	3 (3–4)	6 (3–6)	3 (2–4)	2 (2–3)	4 (2–4)	3 (2–8)
C_min ss_ (ng/mL)	0.929 (1.05, 53.7)	1.80 (1.81, 15.7)	1.85 (2.61, 87.0)	3.33 (3.52, 42.3)	0.724 (0.797, 52.7)	2.57 (2.57, 6.17)
AUC_0-τ,ss_ (ng·h/mL)	37.7 (38.9, 30.5)	64.6 (65.9, 23.8)	79.3 (88.4, 56.0)	153 (153, 4.06)	27.2 (29.0, 42.7)	98.7 (99.1, 9.48)
t_1/2_ (h)^b^	96.9 (52.3)	61.5 (170)	122 (95.9)	69.6 (46.4)	97.1 (45.0)	63.1 (16.1)
CL_ss_/F (L/h)	19.9 (20.6, 31.5)	23.2 (23.7, 26.0)	18.9 (21.1, 55.1)	19.6 (19.6, 4.15)	18.4 (19.5, 33.8)	15.2 (15.3, 10.1)
DCAR						
C_max,ss_ (ng/mL)	0.252 (0.262, 32.0)	0.582 (0.654, 56.4)	0.814 (0.917, 60.0)	1.27 (1.28, 13.4)	0.352 (0.369, 31.0)	1.46 (1.56, 43.3)
T_max_ (h)^a^	6 (6–8)	6 (6–6)	4 (3–4)	4 (4–8)	3 (2–4)	6 (2–24)
C_min ss_ (ng/mL)	0.137 (0.147, 40.8)	0.361 (0.401, 54.4)	0.379 (0.501, 93.2)	0.589 (0.591, 10.0)	0.162 (0.183, 52.7)	0.860 (0.867, 15.5)
AUC_0-τ,ss_ (ng·h/mL)	5.15 (5.32, 29.2)	11.5 (12.6, 51.3)	14.9 (16.9, 63.6)	23.3 (23.6, 21.2)	6.02 (6.38, 38.2)	27.8 (28.5, 27.2)
t_1/2_ (h)^b^	48.1 (38.5)	32.5 (12.8)	50.6 (9.19)	46.1 (22.0)	31.0 (17.3)	59.8 (16.9)
DDCAR						
C_max,ss_ (ng/mL)	0.944 (1.35, 77.2)	3.05 (4.54, 102)	4.23 (5.42, 87.5)	9.24 (9.52, 30.3)	2.06 (2.28, 46.9)	7.16 (7.49, 37.2)
T_max_ (h)^a^	3 (3–24)	3 (0–24)	24 (4–24)	4 (2–24)	1 (0–24)	24 (2–24)
C_min ss_ (ng/mL)	0.758 (1.11, 80.3)	2.51 (3.87, 107)	3.08 (4.24, 97.0)	7.09 (7.65, 48.2)	1.62 (1.80, 45.9)	5.65 (5.93, 36.3)
AUC_0-τ,ss_ (ng·h/mL)	21.2 (29.9, 76.3)	65.7 (98.4, 103)	85.5 (113, 92.9)	201 (209, 36.6)	42.3 (47.2, 46.5)	154 (160, 30.9)
t_1/2_ (h)^b^	467 (50.4)	371 (14.0)	290 (69)	285 (75.7)	298 (79.7)	199 (41.9)

^a^ Median (minimum, maximum).

^b^ Harmonic mean (pseudo SD).

DCAR, desmethyl cariprazine; DDCAR, didesmethyl cariprazine; SD, standard deviation.

Based on mean trough levels, steady state for cariprazine and DCAR appeared to be reached within 1–2 weeks and for DDCAR within 3–5 weeks. At steady state, the rank of exposure in plasma based on AUC was DDCAR > cariprazine > DCAR, which is consistent with data from adults. After the last dose on day 42, mean plasma concentrations of cariprazine and DCAR declined by >90% within 1 week, while mean plasma concentrations of DDCAR declined by ∼50% within 1 week and ∼90% within 6 weeks (Fig. 1).

**FIG. 2.**
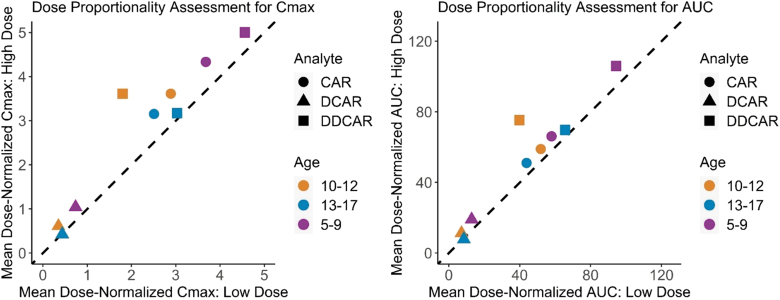
Dose proportionality assessment for steady-state PK parameters of cariprazine and metabolites. Dose proportionality assessment for steady-state pharmacokinetic parameters of cariprazine and metabolites. Pediatric patients 13–17 years old received 1.5 or 3 mg QD. Pediatric patients 10–12 years old received 0.75 or 1.5 mg QD. Pediatric patients 5–9 years old received 0.5 or 1.5 mg QD. CAR, cariprazine; DCAR, desmethyl cariprazine; DDCAR, didesmethyl cariprazine; QD, once daily.

**FIG. 3.**
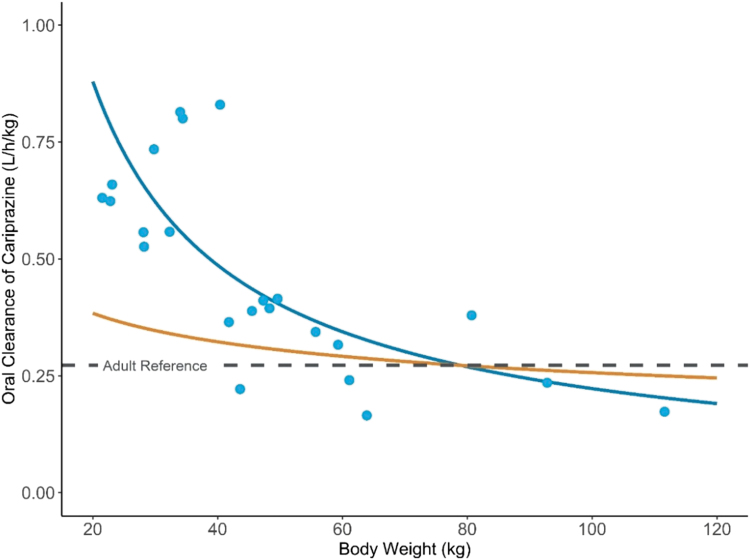
Allometric relationship of oral clearance of cariprazine and pediatric body weight. Allometric relationship between oral clearance of cariprazine (CL_ss_/F) and pediatric body weight. $CL∕F_{child}=21.5L∕h\times {\left(\frac{BW_{child}}{79kg}\right)}^{\theta }$; the optimized allometric exponent (θ=0.15, blue line) is compared against the coventional allometric exponent (θ=0.75, orange line).

Cariprazine, DCAR, and DDCAR steady-state exposures increased approximately in proportion to dose within each of the three age groups (0.5 vs. 1.5 mg for ages 5–9 years; 0.75 vs. 1.5 mg for ages 10–12 years; and 1.5 vs. 3.0 mg for ages 13–17 years) (Fig. 2). The dose-normalized steady-state exposures of all analytes were modestly higher in participants 5–9 years of age compared to older age groups. The geometric mean CL_ss_/F for cariprazine was lower for younger children, and the optimal allometric exponent (θ) that described the power relationship between CL_ss_/F and pediatric body weight was 0.15 (Fig. 3).

### Safety and tolerability

There was no death or SAE reported in this study, and one discontinuation due to an AE of neutropenia: a 5-year-old, African-American male, who had history of benign ethnic neutropenia and no concomitant medication, had an ANC of 1200/μL at screening, followed by a decrease to 900/μL on day 28 and 500 μL on day 42. The caregiver was unreachable until day 56, at which time the ANC was 1700/μL. No sign or symptom of infection was reported. The patient's history of benign ethnic neutropenia provides a possible alternative etiology for this finding. Five other participants with a history of benign ethnic neutropenia did not demonstrate a decrease in ANC.

There was a total of 14 participants (14/25, 56%) who had at least one TEAE reported during the treatment period (Table 6). All reported events were nonserious and mild or moderate in severity. The most frequently reported TEAEs (≥5%) were weight increased (*N* = 8/25, 32%), alanine aminotransferase (ALT) increase (*N* = 2/25, 8%), increased appetite (*N* = 2/25, 8%), dizziness (*N* = 2/25, 8%), agitation (*N* = 2/25, 8%), and nasal congestion (*N* = 2/25, 8%). There was no clinically meaningful imbalance in the number or severity of the reported TEAEs across Cohorts 1–4, except the reported incidence of increased weight was higher for participants 5–9 years of age versus older participants.

**Table 6.** Safety Profile of Cariprazine in Pediatric Patients with Autism Spectrum Disorder

Parameter	Cohort 1 (10–12 years),* N* = 3	Cohort 1 (13–17 years),* N* = 4	Cohort 2 (10–12 years),* N* = 3	Cohort 2 (13–17 years),* N* = 3	Cohort 3 (5–9 years),* N* = 6	Cohort 4 (5–9 years),* N* = 6
≥1 TEAE, *n* (%)	1 (33.3)	3 (75.0)	2 (66.7)	2 (66.7)	4 (66.7)	2 (33.3)
TEAEs occurring in ≥2 patients						
ALT increased	1 (33.3)				1 (16.7)	
Increased appetite					2 (33.3)	
Dizziness	1 (33.3)	1 (25.0)				
Agitation		2 (50.0)				
Nasal congestion	1 (33.3)			1 (33.3)		
Weight increased	1 (33.3)		2 (66.7)		4 (66.7)	1 (16.7)
Potentially clinically significant weight increase						
Weight increase ≥7%,^a^ *n* (%)	1 (33.3)	1 (25.0)	2 (66.7)		6 (100.0)	1 (25.0)
Change from baseline,^a^ mean (SD), kg	2.86 (2.394)		4.03 (3.331)		4.38 (1.489)	0.15 (1.991)

^a^ From baseline to end of study.

TEAE, treatment-emergent adverse event; SD, standard deviation.

A total of 11 patients had a potentially clinically significant weight increase of ≥7% from baseline to the end of the treatment period (Table 6). Overall, baseline mean weight was elevated in this patient population. Z-scores (based on Center for Disease Control and Prevention growth charts) for increases in weight across all cohorts ranged between 0.36 and 0.73 (below 1 standard deviation [SD]) and therefore was considered to generally reflect age- and gender-appropriate growth, However, as with other antipsychotics, mild weight gain is a known risk associated with the use of cariprazine and therefore a causal association between cariprazine and weight increase cannot be excluded in this pediatric population.

In previous animal studies, bilateral cataract and cystic degeneration of the retina was observed in dogs, and retinal degeneration/atrophy was observed in rats, but there was no ocular safety event reported in this study. There was no clinically meaningful ophthalmologic examination finding.

One participant experienced suicidal ideation as per C-SSRS; a further review did not reveal suicidal intent or behavior.

There was no Parkinsonism based on SAS criteria reported in any participant. Two participants experienced akathisia as per BARS criteria. Two participants reported EPS-related TEAEs—one patient had akathisia treated with benztropine, and another patient had restlessness treated with lorazepam, and these TEAEs resolved without leading to discontinuation. The AIMS total score was zero for all participants in Parts A and B at baseline and at all subsequent study visits.

Mean changes in laboratory values of particular interest to the cariprazine safety profile, namely measures of metabolic parameters (glucose and total cholesterol), were generally unremarkable. Two participants had a fasting blood glucose >1.2 × upper limit of normal (ULN); risk factors for these participants included increased body mass index at baseline. Potentially clinically significant changes in liver function tests included ALT increase of >3 × ULN in 3 participants, one of these participants also had an aspartate transaminase increase of >3 × ULN; these participants had slightly elevated (within 3 × ULN) liver enzymes at baseline and laboratory values generally returned to or trended toward normal by the end of the safety follow-up period. No subject met criteria for Hy's Law. No participant had a clinically significant change in ECG parameters.

### Exploratory efficacy

Improvements were seen on all exploratory efficacy endpoints (ABC-I, CGI-S, CgGI-S, CYBOCS-ASD, SRS, and VABS-III) through to the end of the cariprazine treatment period (day 42). Summary statistics for each clinical rating scale are supplied in the Supplementary Data.

**FIG. 4.**
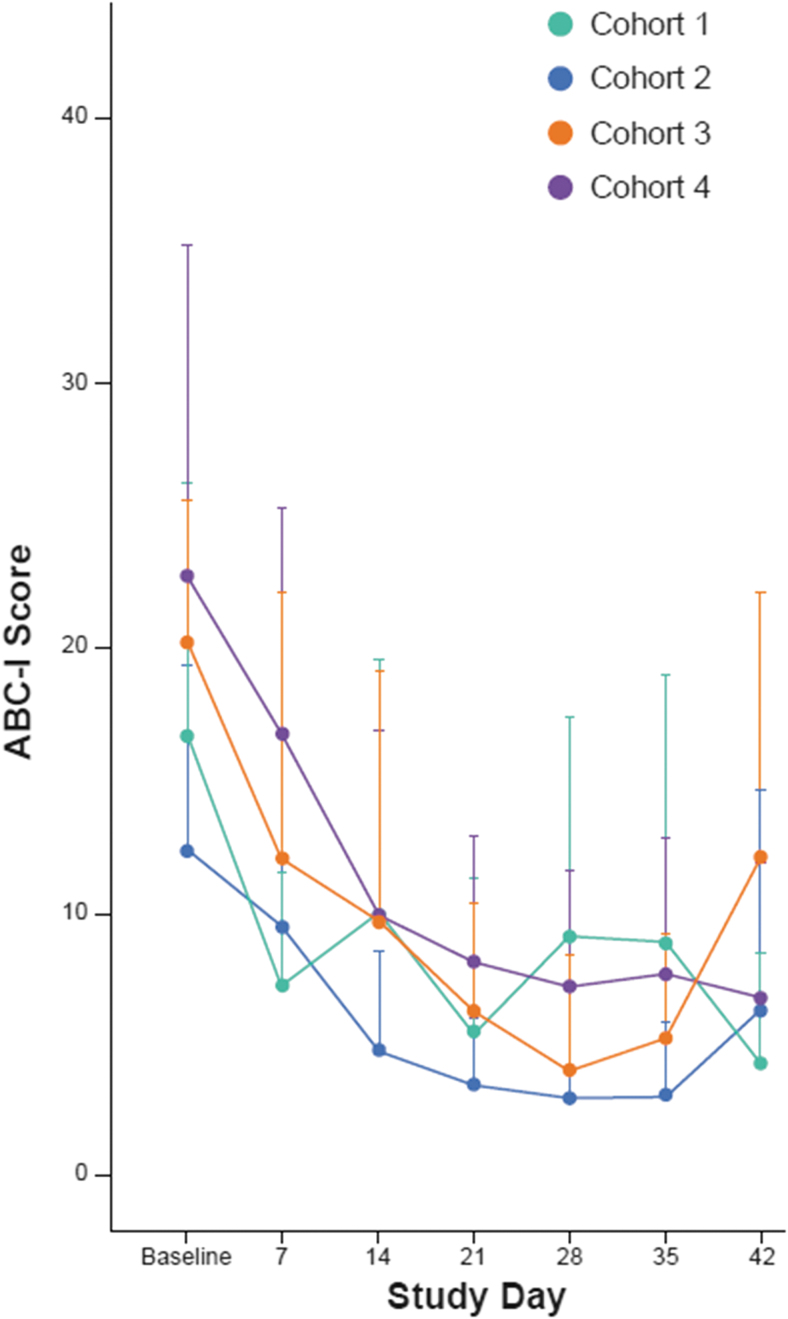
ABC-I scores over 6 weeks of treatment. ABC-I, Aberrant Behavior Checklist-Irritability Subscale.

As a measure of irritability associated with ASD, an improvement in mean ABC-I score was observed in all cohorts at all time points from day 7 onward. Mean (+SD) ABC-I subscale scores by cohort and time point are presented in Figure 4. Across all four cohorts, the mean (SD) change from baseline was −10.5 (11.4) points at the end of the 42-day treatment period.

## Discussion

In this first investigation of cariprazine as an oral solution formulation in pediatric patients with ASD, cariprazine was generally well tolerated over 42 days of treatment with titration of doses up to 1.5 mg/day for children 5–9 years of age and up to 3.0 mg/day for children 10–17 years of age. The safety observations in this study were consistent with the known safety profile of cariprazine in adults.

Steady-state PK of cariprazine, DCAR, and DDCAR was characterized at multiple dose levels of the oral solution formulation. In children 10–17 years of age, dose-normalized exposures of all analytes were similar and within the variability observed in pediatric and adult patients previously studied with the capsule formulation. T_max_ with the oral solution formulation in this study was not appreciably different from T_max_ with the capsule formulation in a previous study (Riccobene et al. 2022). The dose-normalized steady-state exposures of all analytes were modestly higher in participants 5–9 years of age compared to older age groups.

A trend for improvement in ABC-I score was observed across all cohorts to the end of treatment (day 42) and was sustained to the end of the follow-up period (day 84). There was also improvement on other exploratory efficacy endpoints (CGI-S, CgGI-S, CYBOCS-ASD, SRS, and VABS-III). The efficacy results of this study should be interpreted cautiously, since the open-label nature of the study confounds the clinical rating scales. The result warrants investigation of cariprazine for the treatment of irritability in pediatric participants with ASD in a placebo-controlled study.

The antipsychotics risperidone and aripiprazole are indicated for the treatment of irritability associated with autistic disorder in participants 5–17 years of age (6–17 years of age for aripiprazole and 5–16 years of age for risperidone); this includes symptoms of aggression toward others, deliberate self-injuriousness, temper tantrums, and quickly changing moods. Based on the known pharmacological (i.e., receptor binding and behavioral) profile of cariprazine and its similarity to aripiprazole, cariprazine could have an effect on ASD-associated irritability. Furthermore, because of its dopamine D_3_ receptor partial agonist activity, differentiated profile in preclinical ASD-related models, as well as clinical efficacy in the treatment of negative symptoms of schizophrenia (e.g., alogia, ambivalence, and anhedonia), cariprazine could have an effect on similar (core) symptoms of ASD. Consistent with this hypothesis, improvement on SRS and adaptive behavior (VABS-III) was noted in this open-label study (Supplementary Data).

Based on this review of safety data with the oral solution formulation, the nature and frequency of safety observations in pediatric subjects 5–17 years of age were generally consistent with the known safety profile of cariprazine capsule formulation in adults, with no new safety concern for cariprazine identified.

This pediatric PK study of cariprazine contributes meaningful data to inform pediatric dose selection down to 5 years of age for future efficacy studies. In contrast to what would be expected when scaling drug clearance according to the 3/4 rule of allometry, the oral clearance in children was within the variability observed in adults and was dramatically higher than the adult reference clearance on a per kg basis (Fig. 3). The result suggests the pediatric doses (mg) required to match PK exposures to the adult exposures in similar disease states may be near the adult fixed doses, despite differences in body weight. Similar observations have been noted for other oral compounds recently under pediatric development, including tofacitinib (Ruperto et al. 2017), cobimetinib (Trippett et al. 2022), and erlotinib (Reddick et al. 2019), which are mainly metabolized by CYP3A4.

## Conclusions

Cariprazine was generally well tolerated in pediatric patients with ASD at doses up to 3 mg QD (13–17 years) and 1.5 mg QD (5–12 years). Results will inform the selection of appropriate pediatric doses for subsequent studies to evaluate efficacy in ASD.

## Clinical Significance

This study evaluated the PK, safety, and tolerability of cariprazine as an oral solution formulation in pediatric patients with ASD 5–17 years of age and examined efficacy as an exploratory outcome. Similar to previous studies, cariprazine was generally well tolerated among pediatric participants. Based on matching exposures of cariprazine and its metabolites, children younger than 12 years may have only modestly lower dosing requirements (mg) when compared to adults with similar disorders. Symptom improvement from baseline was observed throughout the treatment period with doses up to 1.5 mg QD for children 5–12 years of age and up to 3 mg QD for children 13–17 years of age. These findings support further investigation of cariprazine for pediatric patients with ASD.

## Supplementary Material

Supplemental data
