# Supplementary material for: Cariprazine in Pediatric Patients with Autism Spectrum Disorder: Results of a Pharmacokinetic, Safety and Tolerability Study
Source: J Child Adolesc Psychopharmacol. 2023 Aug 16;33(6):232–42. doi: 10.1089/cap.2022.0097 (PMC10458368; doi:10.1089/cap.2022.0097)
Supplement: Supplemental data [file Supp_DataS1.docx]

# Exploratory Efficacy Endpoints

Table S1. Aberrant Behavior Checklist-Irritability Subscale (ABC-I) Score as Mean (SD) for Each Visit from Baseline to Week 12

|  | **ABC-I Score: Mean (SD)** | | | |
| --- | --- | --- | --- | --- |
|  | **Cohort 1** | **Cohort 2** | **Cohort 3** | **Cohort 4** |
| Baseline | 16.7 (9.60) | 12.3 (7.09) | 20.2 (5.42) | 22.7 (12.58) |
|  | N= 7 | N=6 | N=6 | N=6 |
| Day 7 | 7.3 (4.23) | 9.5 (7.01) | 12.0 (10.08) | 16.7 (8.64) |
|  | N= 7 | N=6 | N=6 | N=6 |
| Day 14 | 10.0 (9.57) | 4.8 (3.71) | 9.7 (9.46) | 9.8 (7.03) |
|  | N= 7 | N=6 | N=6 | N=6 |
| Day 21 | 5.4 (5.91) | 3.5 (2.43) | 6.2 (4.17) | 8.2 (4.71) |
|  | N= 7 | N=6 | N=6 | N=6 |
| Day 28 | 9.1 (8.30) | 3.0 (3.90) | 4.0 (4.38) | 7.2 (4.45) |
|  | N= 7 | N=6 | N=6 | N=6 |
| Day 35 | 8.9 (10.16) | 3.0 (2.83) | 5.3 (3.88) | 7.8 (5.08) |
|  | N= 7 | N=6 | N=6 | N=6 |
| Day 42 | 4.3 (4.27) | 6.5 (8.09) | 12.2 (9.97) | 6.8 (5.19) |
|  | N= 6 | N=6 | N=6 | N=6 |
| Day 49 | 8.4 (9.84) | 3.0 (2.00) | 8.2 (8.61) | 4.0 (2.65) |
|  | N= 5 | N=6 | N=6 | N=3 |
| Day 56 | 5.0 (9.59) | 4.0 (3.85) | 11.0 (9.34) | 7.3 (4.68) |
|  | N= 5 | N=6 | N=6 | N=6 |
| Day 70 | 6.2 (6.94) | 4.8 (3.60) | 9.2 (6.71) | 13.3 (4.73) |
|  | N= 5 | N=6 | N=6 | N=3 |
| Day 84 | 4.2 (6.72) | 3.5 (2.07) | 12.8 (10.08) | 11.7 (3.06) |
|  | N= 5 | N=6 | N=5 | N=3 |

Table S2. Clinical Global Impression of Severity (CGIS) Score as Mean (SD) for Each Visit from Baseline to Week 12

|  | **CGIS Overall Score: Mean (SD)** | | | |
| --- | --- | --- | --- | --- |
|  | **Cohort 1** | **Cohort 2** | **Cohort 3** | **Cohort 4** |
| Baseline | 3.1 (0.69) | 3.2 (0.41) | 3.3 (0.82) | 3.3 (0.52) |
|  | N= 7 | N=6 | N=6 | N=6 |
| Day 7 | 2.6 (0.53) | 2.8 (0.75) | 3.3 (0.52) | 3.2 (0.41) |
|  | N= 7 | N=6 | N=6 | N=6 |
| Day 14 | 2.4 (0.53) | 2.7 (0.52) | 3.2 (0.41) | 2.8 (0.41) |
|  | N= 7 | N=6 | N=6 | N=6 |
| Day 21 | 2.4 (0.53) | 2.5 (0.55) | 2.8 (0.75) | 2.5 (0.55) |
|  | N= 7 | N=6 | N=6 | N=6 |
| Day 28 | 2.6 (0.53) | 2.5 (0.55) | 2.7 (0.82) | 2.2 (0.41) |
|  | N= 7 | N=6 | N=6 | N=6 |
| Day 35 | 2.4 (0.53) | 2.3 (0.52) | 2.5 (0.84) | 2.2 (0.41) |
|  | N= 7 | N=6 | N=6 | N=6 |
| Day 43 | 2.2 (0.41) | 2.3 (0.52) | 2.5 (0.84) | 2.2 (0.41) |
|  | N= 6 | N=6 | N=6 | N=6 |
| Day 49 | 2.4 (0.55) | 2.3 (0.52) | 2.5 (0.84) | 2.0 (0.00) |
|  | N= 5 | N=6 | N=6 | N=3 |
| Day 56 | 2.4 (0.55) | 2.5 (0.55) | 2.7 (0.82) | 2.2 (0.41) |
|  | N= 5 | N=6 | N=6 | N=6 |
| Day 70 | 2.6 (0.55) | 2.5 (0.55) | 3.0 (0.63) | 3.3 (1.15) |
|  | N= 5 | N=6 | N=6 | N=3 |
| Day 84 | 2.6 (0.55) | 2.5 (0.55) | 2.6 (0.89) | 2.7 (0.58) |
|  | N= 5 | N=6 | N=5 | N=3 |

**Clinical Global Impression-Severity Overall (CGIS-Overall):**

The CGIS-Overall (adapted from Guy 1976) is a clinician-rated scale that measures the overall severity of a participant’s illness in comparison with the severity of other participants the clinician has observed. The clinician rated the participant on a 5-point scale (from None to Very severe) based on this question: “Considering your clinical experience with this population, how would you rate the current severity of the patient’s autism spectrum disorder?”

Table S3. Caregiver Global Impression of Severity (CgGIS) Score as Mean (SD) for Each Visit from Baseline to Week 12

|  | **CgGIS Overall Score: Mean (SD)** | | | |
| --- | --- | --- | --- | --- |
|  | **Cohort 1** | **Cohort 2** | **Cohort 3** | **Cohort 4** |
| Baseline | 3.3 (0.76) | 2.8 (0.41) | 3.5 (0.84) | 3.7 (0.52) |
|  | N= 7 | N=6 | N=6 | N=6 |
| Day 7 | 2.6 (0.53) | 2.5 (0.55) | 3.3 (0.52) | 3.5 (0.55) |
|  | N= 7 | N=6 | N=6 | N=6 |
| Day 14 | 2.9 (0.69) | 2.5 (0.55) | 3.3 (0.52) | 2.8 (0.41) |
|  | N= 7 | N=6 | N=6 | N=6 |
| Day 21 | 2.6 (0.53) | 2.5 (0.55) | 2.8 (0.75) | 2.5 (0.55) |
|  | N= 7 | N=6 | N=6 | N=6 |
| Day 28 | 2.9 (0.38) | 2.7 (0.52) | 2.5 (0.84) | 2.2 (0.41) |
|  | N= 7 | N=6 | N=6 | N=6 |
| Day 35 | 2.9 (0.69) | 2.5 (0.55) | 2.5 (0.84) | 2.2 (0.41) |
|  | N= 7 | N=6 | N=6 | N=6 |
| Day 43 | 2.5 (0.55) | 2.7 (0.52) | 2.7 (0.82) | 2.2 (0.41) |
|  | N= 6 | N=6 | N=6 | N=6 |
| Day 49 | 2.8 (0.45) | 2.7 (0.52) | 2.5 (0.84) | 2.0 (0.00) |
|  | N= 5 | N=6 | N=6 | N=3 |
| Day 56 | 2.6 (0.89) | 2.5 (0.55) | 2.5 (0.84) | 2.2 (0.41) |
|  | N= 5 | N=6 | N=6 | N=6 |
| Day 70 | 2.8 (0.45) | 2.5 (0.55) | 3.0 (0.63) | 3.3 (1.15) |
|  | N= 5 | N=6 | N=6 | N=3 |
| Day 84 | 2.2 (0.84) | 2.5 (0.55) | 2.6 (0.89) | 2.7 (0.58) |
|  | N= 5 | N=6 | N=5 | N=3 |

**Caregiver Global Impression-Severity Overall (CgGIS-Overall):**

The CgGIS-Overall was completed by the parent/caregiver to rate the overall severity of the child’s ASD on the same 5-point scale as CGIS-Overall based on this question: “How would you rate the current severity of your child’s autism spectrum disorder?”

Table S4. Children’s Yale-Brown Obsessive Compulsiveness Scale Modified for ASD (CYBOCS-ASD), Social Responsiveness Scale (SRS), and Vineland Adaptive Behavior Scale (VABS-III) Scores as Mean (SD) for Baseline and Following 6 Weeks of Treatment with Cariprazine

|  | **Cohort 1** | **Cohort 2** | **Cohort 3** | **Cohort 4** |
| --- | --- | --- | --- | --- |
|  | **CYBOCS-ASD Score: Mean (SD)** | | | |
| Baseline | 15.3 (2.29) | 11.3 (1.75) | 14.7 (2.80) | 14.0 (2.10) |
|  | N= 7 | N=6 | N=6 | N=6 |
| Day 43 | 9.3 (2.07) | 9.0 (1.67) | 10.0 (4.86) | 6.5 (1.87) |
|  | N= 6 | N=6 | N=6 | N=6 |
|  | **SRS Total Score: Mean (SD)** | | | |
| Baseline | 81.7 (9.98) | 84.2 (6.15) | 79.0 (13.21) | 77.7 (12.37) |
|  | N= 7 | N=6 | N=6 | N=6 |
| Day 43 | 62.3 (4.72) | 79.8 (9.37) | 72.2 (12.97) | 61.8 (16.69) |
|  | N= 6 | N=6 | N=6 | N=6 |
|  | **VABS-III Overall Score: Mean (SD)** | | | |
| Baseline | 212.0 (59.33) | 233.3 (33.15) | 243.3 (48.71) | 227.3 (50.44) |
|  | N= 7 | N=6 | N=6 | N=6 |
| Day 43 | 247.7 (41.89) | 255.2 (31.25) | 269.0 (53.51) | 257.3 (38.91) |
|  | N= 6 | N=6 | N=6 | N=6 |

**Children’s Yale-Brown Obsessive Compulsive Scale: Modified for Autism Spectrum
Disorder (CYBOCS-ASD):**The CYBOCS-ASD is a semi-structured interview designed to measure the severity of repetitive behaviors in children and adolescents with ASD. It is a modified version of the CYBOCS, which was designed to measure obsessive-compulsive symptoms in children and adolescents. The primary informant was the parent/primary caregiver. Using a revised repetitive behavior checklist (the rationale for which is described in Scahill et al 2014), the interviewer begins by asking the parent/caregiver about past and present repetitive behaviors (categories include Hoarding/Ritualistic behavior, Sensory-Motor and Arranging, Insistence on Routines and Self-Injurious Behaviors, Stereotypy, and Restricted interests). Next, a target symptom list including the 4 most troublesome behaviors was established by the interviewer. The severity of each target behavior was rated (on a scale of 0-4) for the following 5 items: Time Spent, Interference, Distress, Resistance and Degree of Control. Two additional items, Repetitive Behavior-Free Interval and Peculiarity were also rated but not included in the total score. Using all available information, the interviewer rated each item using the time frame of the past week.

**Social Responsiveness Scale, 2nd Edition (SRS-2):**
The SRS-2 is a 65-item rating scale that measures the severity of autistic symptomatology as a
quantitative trait, among children clinically affected by autism spectrum disorder as well as
among children in the general population. It is particularly useful for characterizing milder autistic syndromes that lie at the boundary between the normal population distribution and
clinical presentations (Constantino et al 2009).

**Vineland Adaptive Behavior Scale, 3rd Edition (VABS-III):**
The VABS-III evaluates children's personal and social sufficiency in a semi-structured interview with a primary caregiver (Sparrow et al 2016). A trained evaluator used the interview format of the VABS-III to conduct the assessment. The VABS-III is a measure of adaptive behavior for pediatric and adult populations with intellectual and developmental disabilities aged 0 to 90 years. This instrument assesses four areas of adaptive behavior: Communication, Daily Living Skills, Socialization, and Motor Skills. The VABS provides another opportunity to evaluate the effects of cariprazine on the communication and socialization symptoms of ASD.

References:

- Constantino JN, Abbacchi AM, LaVesser PD, Reed H, Givens L, Chiang L, et al. Developmental course of autistic social impairment in males. Dev Psychopathol. 2009;21:127-138.
- Guy W. (1976) Clinical Global Impressions; Abnormal Involuntary Movement Scale. In: ECDEU Assessment Manual for Psychopharmacology. DHEW Publication No. (ADM) 76-338 ed. Rockville, MD: US Department of Health, Education and Welfare, Public Health Service, Alcohol, Drug Abuse and Mental Health.
- Scahill L, Dimitropoulos A, McDougle CJ, Aman MG, Feurer ID, McCracken JT, et al. Children’s Yale-Brown Obsessive Compulsive Scale in Autism Spectrum Disorder: component structure and correlates of symptom checklist. J Am Acad Child Adolesc Psychiatry. 2014;53(1):97-107.e1.
- Sparrow SS, Cicchetti DV, Saulnier CA. Vineland Adaptive Behavior Scales, Third Edition (Vineland-3). 2016. San Antonio, TX: Pearson.
